# Dysbindin-1 Involvement in the Etiology of Schizophrenia

**DOI:** 10.3390/ijms18102044

**Published:** 2017-09-22

**Authors:** Haitao Wang, Jiangping Xu, Philip Lazarovici, Wenhua Zheng

**Affiliations:** 1 Department of Neuropharmacology and Drug Discovery, School of Pharmaceutical Sciences, Southern Medical University, Guangzhou 510515, China; wht821@smu.edu.cn (H.W.); jpx@smu.edu.cn (J.X.); 2 School of Pharmacy Institute for Drug Research, Faculty of Medicine, The Hebrew University of Jerusalem, Jerusalem 91120, Israel; philipl@ekmd.huji.ac.il; 3 Faculty of Health Sciences, University of Macau, Taipa, Macau 999078, China; 4 Zhuhai UM Science & Technology Research Institute, Zhuhai 519080, China

**Keywords:** schizophrenia, dysbindin-1, susceptibility gene, neurotransmitter release, neurite outgrowth

## Abstract

Schizophrenia is a major psychiatric disorder that afflicts about 1% of the world’s population, falling into the top 10 medical disorders causing disability. Existing therapeutic strategies have had limited success on cognitive impairment and long-term disability and are burdened by side effects. Although new antipsychotic medications have been launched in the past decades, there has been a general lack of significant innovation. This lack of significant progress in the pharmacotherapy of schizophrenia is a reflection of the complexity and heterogeneity of the disease. To date, many susceptibility genes have been identified to be associated with schizophrenia. *DTNBP1* gene, which encodes dysbindin-1, has been linked to schizophrenia in multiple populations. Studies on genetic variations show that *DTNBP1* modulate prefrontal brain functions and psychiatric phenotypes. Dysbindin-1 is enriched in the dorsolateral prefrontal cortex and hippocampus, while postmortem brain studies of individuals with schizophrenia show decreased levels of dysbindin-1 mRNA and protein in these brain regions. These studies proposed a strong connection between dysbindin-1 function and the pathogenesis of disease. Dysbindin-1 protein was localized at both pre- and post-synaptic sites, where it regulates neurotransmitter release and receptors signaling. Moreover, dysbindin-1 has also been found to be involved in neuronal development. Reduced expression levels of dysbindin-1 mRNA and protein appear to be common in dysfunctional brain areas of schizophrenic patients. The present review addresses our current knowledge of dysbindin-1 with emphasis on its potential role in the schizophrenia pathology. We propose that dysbindin-1 and its signaling pathways may constitute potential therapeutic targets in the therapy of schizophrenia.

## 1. Introduction

Schizophrenia is an idiopathic mental illness occurring in 0.5–1% of the general population [1]. The clinical symptoms of this disorder include auditory hallucinations, delusions, disorganized speech, abnormal motor behavior, cognitive deficits and other behavioral symptoms [1,2]. Schizophrenia-like symptoms also include negative symptoms, such as reduced response to daily and social activities (motivation), depressive-like emotion or diminished expression of pleasure [3]. These symptoms impair patients’ daily functioning, and can be disabling [4]. Despite the efforts to develop effective interventions, drugs and psychosocial therapies, an efficient treatment of schizoaffective disorder is not yet available. Most of the antipsychotic drugs control some schizophrenic symptoms by affecting the dopamine and/or serotonin in the brain, however schizophrenia requires chronic therapy and antipsychotics usually cause intolerable side effects [5]. Failures of investigational new drugs for schizophrenia have left huge unmet medical needs for patients. Given the recent lackluster results, it is imperative that new drug candidates that target pathophysiological alterations that are shared in specific patient populations is becoming increasingly necessary for future investigational new drugs [6].

It is generally accepted that genetic and environmental factors can trigger the disease [7]. In fact, gene-by-environment interactions and epigenetic alterations modified by environmental and social factors are now considered as possible facets of the schizophrenia etiology [8]. A large number of epidemiological studies carried out in family and twin established that the vulnerability to develop schizophrenia is largely genetic in stratified patient populations [9,10]. Family studies found that the lifetime morbid risks of schizophrenia in relatives of patients is 10 times higher than that in relatives of controls [11,12]. Heritability of schizophrenia is around 80–85% and twin studies have indicated 50% chance in monozygotic and 17% chance in dizygotic twin obtaining the diagnosis (if the other twin already has it) [12,13]. It is interesting that the rates of developing schizophrenia were quite low and no difference was observed in the adoptive families of both affected and control groups [12]. These studies clearly indicate the important role of genetic factors in the pathogenesis of schizophrenia. The genetic basis of schizophrenia is complex and genome-wide association studies (GWAS) have identified hundreds of single nucleotide polymorphisms (SNPs) associated with schizophrenia [14].

Many studies have shown that dystrobrevin binding protein-1 (dysbindin-1) is one of the important potential susceptibility genes for schizophrenia [15,16,17,18,19,20,21]. Accumulating evidence shows that the level of dysbindin-1 is reduced in postmortem brains from schizophrenia patients [22,23]. Studies on its neurobiological functions indicate that dysbindin-1 regulates neurotransmitter release, post-synaptic receptor expression and brain development [24,25,26,27,28,29]. Thus, an enhanced understanding of the biological functions and molecular pathways mediated by dysbindin-1 is required to better exploit the therapeutic potential of dysbindin-1 for the treatment of schizophrenia. In this review, we will examine the current understanding and evidence that are proposing dysbindin-1 involvement in schizophrenia and explore its potential as an intervention target for the treatment of schizophrenia.

## 2. Expression of Dysbindin-1 in the Brain and Its Biological Functions

Dysbindin-1 is a protein encoded by *dystrobrevin-binding protein 1* gene (*DTNBP1*), which is located on the short (p) arm of chromosome 6 at position 22.3 [30]. Initially, dysbindin-1 was found to be a component of the dystrophin-associated protein complex (DPC) in skeletal muscle cells. It is believed that dystrophin-1, which links the cell cytoskeleton to the extracellular matrix, contributes to the stability of muscle fibers and, therefore, the loss of dysbindin-1 is probably involved in the pathology of muscular dystrophy [31,32]. DPC is also highly expressed in the brain, in particular the cortex and the hippocampus. As a component of the DPC complex, dysbindin-1 in the central nervous system (CNS) maintains the structure and physical stabilization of neuronal synaptic membrane [33].

Dysbindin-1 is also a part of biogenesis of lysosome-related organelles complex 1 (BLOC-1) [34], which is involved in the biogenesis of specific components, such as melanosomes and platelet-dense granules, of the endosomal-lysosomal system [35]. In the CNS, BLOC-1 subunits co-localized with synaptic vesicles and synaptosomes derived from synaptic endings [36]; therefore, it was proposed that these BLOC-1 subunits control membrane expression and lysosomal delivery of post-synaptic receptors [37]. As a component of BLOC-1, dysbindin-1 is found primarily in axon or synaptic terminals in the striatum, neocortex, cerebellum and hippocampus [32], brain areas affected in schizophrenic patients. BLOC-1 complex contains many proteins, including pallidin, muted, cappuccino, dysbindin, snapin, blosl, blos2 and blos3 [38]. Mutation or deletion of dysbindin-1 subunit is associated with a destabilization of these proteins of the BLOC-1 complex [37], and defects on the dysbindin-1 complex contribute to synaptic and circuit deficits [36]. Moreover, its deficiency affects the expression of post-synaptic neurotransmitter receptors [37,39,40,41], which are involved in schizophrenia pathogenesis.

Dysbindin-1 is an evolutionary conserved protein composed of approximately 350 amino acids and containing two coiled-coil domains [42]. There are three dysbindin-1 isoforms, namely dysbindin-1A, -1B and -1C [42]. All three of these isoforms are highly expressed in neuronal cells, while dysbindin-1A is the longest and major isoform expressed in the brain [28]. Dysbindin-1A and -1B have the same N-termini, whereas dysbindin-1B lacks exon encoding the PEST ((proline (P), glutamic acid (E), serine (S) and threonine (T)) domain in the C-terminal [43]. Dysbindin-1A and -1C have the same C-termini, while dysbindin-1C lacks the N-terminal 81 amino acids [43]. Isoforms 1A and 1B are mainly localized in nucleus, whereas the isoform C is exclusively expressed in the cytosol [42]. Dysbindin-1A is expressed on postsynaptic densities (PSDs), dysbindin-1B is mainly expressed in synaptic vesicles, and dysbindin-1C is expressed both in synaptic vesicles and present in PSDs [28]. Knock down of dysbindin-1 resulted in an imbalance of the dopaminergic system and dysregulation of hippocampal synaptic transmission [26,27,44,45,46], pathological processes of schizophrenia. The levels of both dysbindin-1B and 1C are reduced in the hippocampus of schizophrenic patients, while level of synaptic dysbindin-1A was not affected [36], suggesting that different dysbindin-1 isoforms have different biological functions in schizophrenia. Among the three isoforms, dysbindin-1C was implicated in neurogenesis and neurodevelopment. Deficiency of dysbindin-1C leads to a reduction in mossy cells and delayed maturation of newborn neurons in the adult hippocampus [29]. These data suggest that distinct dysbindin-1 isoforms regulate neurodevelopment and reduced expression dysbindin-1C is probably associated with impairment of adult hippocampal neurogenesis.

## 3. Interaction of Dysbindin-1 with Cellular Proteins

It is widely accepted that that dysbindin-1 interacts with multiple proteins and exhibits different biological functions in different tissues [32,47]. In the CNS, dysbindin-1 and DPC were implicated in the formation and stability of neuronal synapses as well as the regulation of dendritic spine morphogenesis [33]. Schizophrenia is commonly viewed as a neurodevelopmental disorder originating from decreased spine density and impaired synaptic connectivity [48]. Therefore, it is hypothesized that deficiency of dysbindin-1 leads to profound dysfunction in synaptic connectivity, and eventually contributes to the schizophrenia-like pathology [33]. Besides the originally identified interacting protein dystrobrevin, it has been reported that dysbindin-1 interacts with many proteins, such as histone deacetylase 3 (HDAC3) [49], DNA-dependent protein kinase (DNA-PK) [42], nuclear factor-kappa B (NF-κB) [50], disrupted in schizophrenia 1 (DISC1) [51] and snapin [52].

Dysbindin-1 formed a protein complex with HDAC3 in human neuroblastoma cells and in mouse brain. The interaction between dysbindin-1 and HDAC3 occurred in an isoform-specific manner: HDAC3 coupled with dysbindin-1A and -1B, but not -1C. It was also found that dysbindin-1B expression was increased in the nucleus in the presence of HDAC3, and conversely, that the phosphorylation level of HDAC3 increased in the presence of dysbindin-1B [49]. Therefore, it is tempting to propose that dysbindin-1 may regulate gene transcription through an interaction with HDAC3. In patients with psychiatric disorders, histone acetylation is significantly reduced and inhibition of HDAC might be a promising strategy for cognition improvement in schizophrenic patients [53]. In the nucleus, dysbindin-1 forms a protein complex with HDAC3 and DNA-dependent protein kinase (DNA-PK). DNA-PK complex promotes the phosphorylation of both dysbindin-1 and HDAC3 [42]. Among the three isoforms, dysbindin-1A, and -1B localize in the nucleus and interact with HDAC3, while only dysbindin-1B facilitates the phosphorylation of HDAC3 by DNA-PK [49]. Since dysbindin-1A and -1B have different C-termini, it is hypothesized that C-terminal plays a key role in mediating the isoform-specific interaction with HDAC3 [49]. NF-κB is a transcription factor involved in neuronal outgrowth and synaptic plasticity [54]. In schizophrenic patients, the activity of NF-κB is significantly decreased [55], implicating NF-κB in the etiology of schizophrenia. Furthermore, dysbindin-1A is degraded in the nucleus via the ubiquitin-proteasome system and dysbindin-1A amino acids 2-41 at the *N*-terminus are required for this process [50]. Dysbindin-1A has been proved to interact with p65, a subunit of NF-κB, in the nucleus and enhance the transcriptional activity of NF-κB [50]. Considering nuclear-cytoplasmic shuttling property in combination with its nuclear degradation and possible regulation of NF-kappa B activities, it is reasonable to propose an important role for dysbindin-1A during schizophrenia pathogenesis. Moreover, as NF-κB has been linked to neuroinflammatory responses in relation to neurodegeneration [56] and schizophrenia [57], we suggest that dysbindin-1A interaction with the NF-κB signaling cascade is responsible for localized neuroinflammation in higher areas of the brain involved in the behavioral and clinical symptoms of schizophrenia.

**Figure 1:**
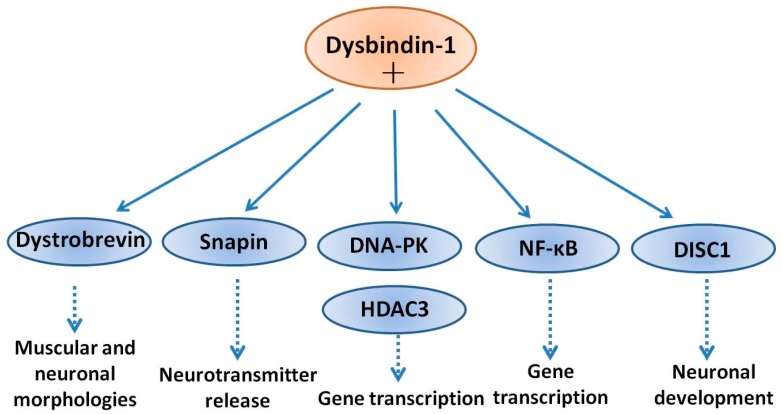
Interaction of dysbindin-1 with cellular proteins. Dysbindin-1 has been shown to associate into complexes with multiple binding partners, in both the cytoplasm and nucleus. Dysbindin-1 binds to α- and β-dystrobrevins and regulates muscular and neuronal morphologies. Dysbindin-1 may also interact with snapin and disrupted in schizophrenia 1 (DISC1), and thereby modulating neurotransmitter release and neurodevelopment, respectively. In the nucleus, dysbindin-1 can form a complex with DNA-dependent protein kinase (DNA-PK) and promote the phosphorylation of histone deacetylase 3 (HDAC3). In addition, interaction of dysbindin-1 with DISC1 may dysregulate neuronal development.

DISC1 is another schizophrenia susceptibility factor playing roles in neuronal development [58]. In neuronal cells, DISC1 forms a complex with dysbindin-1, increases its stability in association with a reduction in ubiquitination and the physical interaction is critical for the process of neurite outgrowth. Furthermore, knockdown of DISC1 or expression of a deletion mutant, effectively decreased the levels of endogenous dysbindin-1. Moreover, the neurite outgrowth defect induced by knockdown of DISC1 was partially reversed by co-expression of dysbindin-1. Taken together, these results indicate that dysbindin-1 and DISC1 form a physiologically functional complex that is essential for neurite outgrowth [59]. Snapin is a binding partner of dysbindin-1 in the brain. Tissue fractionation of whole mouse brains and human hippocampal formations revealed that both dysbindin-1 and snapin are concentrated in tissue enriched in synaptic vesicle membranes and less commonly in postsynaptic densities. Consistent with that finding, localization studies indicated that dysbindin-1 is located in: (i) synaptic vesicles of axonal terminals in the dentate gyrus inner molecular layer and CA1 striatum radiatum; and (ii) postsynaptic densities and microtubules of the dentate neurons and CA1 pyramidal cells [60]. The function of dysbindin-1 in presynaptic, postsynaptic and microtubule locations may all be related to known functions of snapin in regulation of neurotransmitter release [61]. Dysbindin-1 and snapin are components of BLOC-1 and snapin is another binding partner of dysbindin-1 in vitro and in the brain [60]. Deletion in the *DTNBP-1* gene causes reduced level of snapin, which is accompanied by defects of synaptic morphology in hippocampal neurons and schizophrenia-like behaviors in mice [62]. Disruption of dysbindin–snapin complex is supposed to affect microtubule assembly, which could contribute to reductions in the neuronal cell size and reduced dendritic density. All of these morphological alterations were frequently observed in schizophrenia [60]. A scheme of the above interactions of dysbindin-1 with several neuronal proteins is shown in Figure 1.

## 4. Association of Dysbindin-1 with Schizophrenia

Numerous studies have suggested a genetic predisposition to schizophrenia, and many genes, including *DISC1*, catechol-*O*-methyltransferase (COMT), neuregulin 1 (NRG1), and *DTNBP1*, have been identified as candidate susceptibility genes [2,63]. *DTNBP1* is found at chromosomal locus 6p22.3 and mutations on this locus have been linked to schizophrenia [64]. Several single SNPs of *DTNBP1* were suggested to influence multiple psychiatric phenotypes in schizophrenic patients. For example: (i) SNP rs1997679 and SNP rs9370822 were proven to be associated with visual hallucination [65]; (ii) SNP rs4236167 was associated with auditory hallucination [65]; (iii) SNP rs9370822 and SNP rs9370822 were found associated with olfactory hallucinations [65]; (iv) SNP rs909706, rs760761 and rs1018381 were associated with attention [66,67]; (v) SNP rs2619522 was correlated with hippocampal and prefrontal grey matter volumes in schizophrenic patients [68]; and (vi) SNP rs9370822 affected glutamatergic or dopaminergic neurotransmission and has been found to be associated with a number of psychiatric conditions including schizophrenia [69]. In 2014, Schizophrenia Working Group of the Psychiatric Genomics Consortium reported a multi-stage schizophrenia genome-wide association study of more than 150,000 people and found 108 schizophrenia-associated genetic loci [70]. Many of these were involved in dopamine receptor subtype 2 (D2R) and glutamatergic neurotransmission, findings consistent with pathophysiological hypotheses of schizophrenia [70]. Interestingly, there are several works reporting in animal studies that genetic disruption of dysbindin-1 can alter dopaminergic and glutamatergic neurotransmission [46,71,72,73,74]. Therefore, mutations in Dysbindin gene causing partial or full loss of function, may represent as a direct genetic bridge between these two neurotransmitter systems [74]. Statistical evidence for genetic association is strongly supported by altered *DTNBP1* gene expression in schizophrenic brain.

Postmortem studies have indicated that mRNA and protein expression of dysbindin-1 is decreased in the brains of schizophrenic patients [75]. Talbot et al. found that presynaptic dysbindin-1 was significantly reduced in the hippocampal formation in schizophrenic populations [22]. The reduction of dysbindin-1 protein was related to glutamatergic alterations and was proposed to contribute to the cognitive deficits in schizophrenia [22]. Weickert et al. found that patients with schizophrenia had a significant reduction of dysbindin mRNA levels in the dorsolateral prefrontal cortex and midbrain [23]. Therefore, considering the reduced dysbindin-1 expression in schizophrenic patients, together with the data from SNPs of *DTNBP1* gene mutations, we propose that dysbindin-1 is an etiologic factor in schizophrenia.

## 5. Dysbindin-1 Mutation Links to Schizophrenia-Like Behaviors

Dysbindin-1 knockout mice offer an ideal tool to study the biological and pathological roles of dysbindin-1 in the brain and development of schizophrenia. Sandy (sdy) mice are dysbindin-1 knockout mice generated in the Jackson Laboratory. These mice have a deletion mutation occurring spontaneously in the inbred DBA/2J mice on the gene encoding dysbindin-1 (*DTNBP1*) [76]. Dysbindin-1 deletion has no effect on body weight, appearance, sensory-motor reflexes and neuromuscular strength, while Sdy mice displayed decreased locomotor activity and deficits in social interaction [44]. This phenotype is possibly due to a decreased motivation to explore, which is highly related to the negative symptoms of schizophrenia. In addition to hypo-locomotor activity, Sdy mice also showed cognitive losses, including deficit of long-term memory retention and impaired working memory [77], representative neurobiological traits observed in patients with schizophrenia [78]. The original Sdy mice were based on DBA/2J genetic background. This background itself is characterized by locomotor and memory deficits that may confound the explanation of phenotypes observed in this mouse [79]. Hence, the Sdy mutant mice were thereby produced from the C57BL/6J background (dys^−/−^). Consistently, dys^−/−^ mice also showed displayed clear deficits in spatial learning and memory using the Morris water maze and T-maze tests [46,71]. However, dys^−/−^ mice are hyperactive in an open-field test [71]. In addition, abnormal pre-pulse inhibition (PPI) of an acoustic startle stimulus is usually observed in individuals with schizophrenia [80]. Similar to this observation, dys^−/−^ mice showed higher acoustic startle reactivity to the 120-dB stimulus [46], indicating that reduced dysbindin-1 levels in mice, mimicked the increased reactivity to stressful events seen in patients. These results support the association of dysbindin-1 to psychosis in humans. Mechanistic studies showed that dysbindin-1 mutation in mice dysregulated pre- and post-synaptic glutamatergic transmission and the expression of the *N*-methyl-d-aspartic acid (NMDA) receptors was significantly decreased [81]. Dys^−/−^ mice also revealed deletion of dysbindin-1, reduced expression of Ca^2+^/calmodulin-dependent protein kinase II (CaMKII) in medial prefrontal cortex [46] and enhanced cell surface recycling and insertion of D2R into the cell membrane [41], processes which may strengthen D2R-mediated signaling. Collectively, the results from dysbindin-1 knockout animals support the view that dysbindin-1 may increase the risk for schizophrenia by disrupting glutamate and dopamine-related mechanisms regulating cortical function and neuronal excitability.

## 6. Regulation by Dysbindin-1 of Neurotransmitter Receptors

The dysfunction of dopamine is a well-established working hypothesis of schizophrenia [82]. Recently, a “dual topographic dysregulation” of dopamine alteration has been proposed in the reevaluation of the DA hypothesis of schizophrenia [82,83]. While within the striatum, especially in the rostral caudate, the release and synthesis of dopamine is excessive, outside of the striatum, the release of dopamine is in deficit in most brain regions, (cortex, hippocampus, and midbrain) [82,83]. In addition, striatal-cortical connections are significantly disrupted in patients with schizophrenia. In this context is important to stress that abnormal striatal connectivity specifically correlates with severity of positive symptoms and lower density of extra-striatal D2R within the same individual [84]. On the other hand, cumulative evidences have shown that the core pathophysiology of schizophrenia might involve dysfunction of glutamate [2]. Summarizing, increasing molecular evidences support a dual role of dysbindin-1 in dopamine and glutamate signaling.

Several studies have examined the regulatory role of dysbindin-1 in dopaminergic transmission: (i) dysbindin-1 is a component of BLOC-1 involved in intracellular protein trafficking and synaptic homeostasis [39]; (ii) mutation of dysbindin-1 caused impaired trafficking of D2R and increased expression of D2R on cell surface of brain cortical neurons [41]; (iii) decreased expression of dysbindin-1 did not affect dopamine D1 receptors (D1R) [41]; (iv) decreased *DTNBP1* mRNA using siRNA transfection increased the expression of D2R in both SH-SY5Y neuroblastoma cells and in primary cultured cortical neurons [41]; and (v) in dysbindin-1-deficient mice, pyramidal neurons in medial prefrontal cortex were more sensitive to D2R agonist-induced behaviors, while they were less sensitive to D2R antagonist [46].

These findings indicate that dysbindin-1 regulates the expression of D2R on neuronal cell surface as well as modulates D2R-related behaviors. Moreover, dysbindin-1 also affects D2R-mediated signaling. Dysbindin-1 deficiency in the brain reduced Ca^2+^/calmodulin-dependent protein kinase II (CaMKII) expression and signaling in medial prefrontal cortex, while chronic D2R agonist treatment reversed the changes in signaling [46]. Additionally, Dysbindin-1 reduced dopamine-induced adenylate cylase/cAMP signaling and phosphorylation of protein kinase B/glycogen synthase kinase-3β (Akt/GSK3β) and extracellular signal-regulated kinase1/2 (ERK 1/2) [85]. Hence, reduced expression of dysbindin-1 in the brain of schizophrenic patients may decrease dopaminergic signaling, supporting its link to the etiology of schizophrenia.

**Figure 2:**
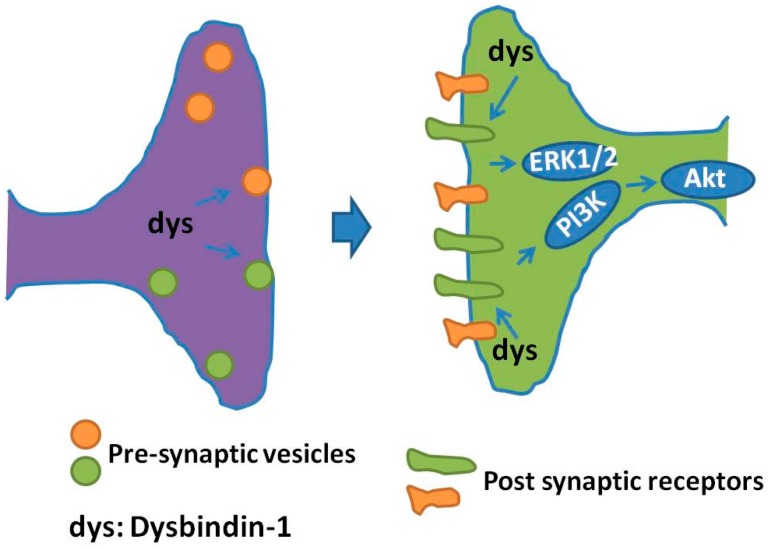
Regulation of dysbindin-1 on pre-synaptic vesicles and post-synaptic receptors. In the pre-synaptic terminals, dysbindin-1 regulates synaptic vesicle transport and release. In the post synaptic terminals, dysbindin-1 regulates the trafficking of neurotransmitter receptors, such as dopaminergic receptors, and affects the expression of these receptors on neuronal cell surface. Dysbindin-1 also modifies protein kinase B (Akt) and extracellular signal-regulated kinase1/2 (ERK1/2) signaling through acting on G protein receptor-induced adenylate cyclase recruitment and cAMP production.

Besides dopamine receptors, dysbindin-1 may impact glutamate receptor(s) and their signaling: (i) decreased NMDA-evoked currents and NMDA receptor subunit 1 expression was observed in prefrontal pyramidal neurons in dysbindin-1 mutant mice [81], indicating that dysbindin-1 deficiency down regulates both the expression and function of NMDA receptors; (ii) hippocampal slices from dysbindin mice exhibited an enhanced long term synaptic potentiation (LTP), a process directly correlated with elevated neuronal cell surface expression of NMDA type subunit 2A [86]; (iii) dysbindin-1 deficiency caused impairment in hippocampal synaptic plasticity and hippocampal-dependent memory [87], functions in which both mGluRI and NMDA receptor are involved [73]; and (iv) dysbindin-1 is expressed in forebrain glutamatergic neurons and dysbindin mutants showed impairments of prefrontal cortical glutamatergic circuits [88]. Cumulatively, these findings propose a role for dysbindin-1 in NMDA system, which is supposed to affect cognitive impairments of schizophrenic patients [87]. A schematic of the regulation of pre-synaptic vesicles and post-synaptic receptors regulated by dysbindin-1 is presented in Figure 2.

## 7. Dysbindin-1 Regulation of Neurite Outgrowth

Neurodevelopmental disturbance may contribute to the pathogenesis of schizophrenia and schizophrenia had also been viewed as a developmental encephalopathy [89]. This hypothesis is further supported by the fact that many of candidate genes in schizophrenia have also been clearly shown to be linked to the neurodevelopment process [89]. The sprouting and elongation of neurites (neurite outgrowth) is an initial critical process in the early stage of neurodevelopment of the nervous system [90]. Dysbindin is expressed embryonically and the level of dysbindin is higher during embryonic and early postnatal stage than that in adulthood [24]. The average level of dysbindin-1 in hippocampus and cerebral cortex from postnatal day 1 mice was three times higher than that from postnatal 45 days aged mice [24]. The role of dysbindin-1 in neurite outgrowth has been extensively documented. Primary cultures of dysbindin-1-deficient neurons display neurite extension defects: reduced number of neurites and shorter length compared with wild type neurons [24]. In line with these findings, knockdown of dysbindin-1 in rat hippocampal neurons using siRNA caused abnormally elongated, immature-dendritic protrusions [33]. Consistent with these findings, siRNA-mediated knockdown of dysbindin-1 in human neuroblastoma SH-SY-5Y cells, caused shorter neurites and abnormal organization of the actin cytoskeleton at their growth cone [91]. Furthermore, in vitro cultured hippocampal neurons, derived from dysbind-1 knockout mice, showed morphological abnormalities of the actin cytoskeleton on growth cones [91]. Together, these results indicate that deficiency of dysbindin-1 might cause subtle defects during neurodevelopment, neural network organization, and activity. Dysbindin-1 may also affect pathologically neurite outgrowth through interacting with other proteins. For example, both dysbindin-1 and DISC1 are susceptibility factors for schizophrenia [2,59]. DISC1 has been reported to be an essential component regulating neurite outgrowth during neuronal differentiation [92]. In the CNS, DISC1 forms a complex with dysbindin-1 and their physical interaction is essential for normal neurite outgrowth [59]. Moreover, dysbindin-1 may also facilitate neurite outgrowth indirectly through regulating the transcriptional activity of p53 [25], which is a tumor suppressor and involved in neurodevelopment as well [93]. These studies stress the important role of dysbindin-1 in the process of neurite outgrowth. Therefore, further studies are needed to shed light on interactions between signaling pathways regulating neurite outgrowth and dysbindin-1.

## 8. Dysbindin-1 Is Required for Diverse Presynaptic and Postsynaptic Mechanisms

Since dysregulation of presynaptic and postsynaptic mechanisms of neurotransmitter release could contribute to the etiology of schizophrenia [94], defining the protein interaction networks for dysbindin-1 in the neuronal synapse will help understanding its functions. Dysbindin-1 is localized in the synapse and has multiple essential roles in presynaptic and postsynaptic pharmacology [39]. In recent years, using biochemical methods such as immunoprecipitation, mass spectrometry and protein expression analysis, a number of interactions between dysbindin-1 and an array of other neuronal proteins were identified. In the presynaptic terminal, dysbindin-1 is involved in different aspects of vesicle trafficking processes [95]. For example, dysbindin-1 participates in synaptic vesicle biogenesis and cargo sorting through interacting with BLOC1 and adaptor-related protein complex-3 (AP3) [95], dysbindin-1 regulates vesicle trafficking through interaction with actin and tubulin-based cytoskeletons, such as dynactin and tubulin/actin proteins [95]. Moreover, dysbindin-1 is involved in membrane targeting and vesicle tethering by binding and interacting with exocyst [95]. At pre-synaptic terminals, dysbindin is co-localized with Munc18-1, a neuron-specific protein essential for the exocytosis of synaptic vesicles [96], modulating synaptic vesicle fusion and neurotransmitter release [97]. These findings suggest that impairment of the presynaptic vesicle functions regulated by dysbindin-1 may be a pathogenic mechanism in schizophrenia.

Recently, Gokhale et al. applied quantitative mass spectrometry to identify the proteomics of neuronal cells with dysbindin-1 deficiency [98]. Both dysbindin-1 and actin-related protein 2/3 (Arp2/3) complex subunits localized to presynaptic and postsynaptic terminals in neuronal cells [60,98]. It is known that synaptic terminals’ dysbindin and Arp2/3 complex are involved in regulation of structural plasticity of dendritic spines, actin protein polymerization, and expression of neurotransmitter receptors [99,100,101]. Since the expression of Arp2/3 complex is reduced in dysbindin-deficient cells, it is assumed that interaction of dysbindin-1 with the Arp2/3 complex modulates presynaptic plasticity and adaptive synaptic responses [98]. These findings propose that dysbindin-1 is necessary for neuronal synaptic plasticity. A proteome-wide search for expression of proteins which are affected by dysbindin/BLOC-1 deficiency in neuronal cells showed that expression of 224 proteins is altered [102]. Annotation of these proteins to neuronal functions indicates that in a majority they are involved in neurotransmitter vesicle fusion and synaptic plasticity [102]. Additional quantitative proteomic studies have identified changes in expression of proteins and polypeptides sensitive to dysbindin/BLOC-1 loss of function, including: (i) the BLOC-1 subunits, such as Bloc1s1-5 and snapin; (ii) dynactin complex, such as alpha-centractin and dynactin 2; (iii) exocyst complex, such as exocyst 3 and exocyst 4; (iv) tubulin/actin associated proteins, such as actin alpha 1 and tubulin alpha 1b; (v) AP3 complex, such as adaptor-related protein complex-3B1 and -2; (vi) vesicular transport/trafficking associated/fusion apparatus, such as adaptor protein 2A1, the vesicle associated membrane protein 7, syntaxin-binding protein 1 and 5; (vii) proteasome subunits, such as proteasome modulator 9 and proteasome subunit alpha type 4; and (viii) other proteins, such as the copper-transporting P-type adenosine triphosphatase (ATP7A), the *N*-ethymaleimide-sensitive factor, annexin A2, syntaxin 7 and 17, synaptosomal-associated protein 25 and family with sequence similarity 91 member A1 [95,98,102,103,104]. Future investigations on these dysbindin-1-interacting proteins are expected to expand dysbindin-1 neuronal functions and provide alternative, additional molecular targets for schizophrenia susceptibility.

## 9. Summary, Conclusions and Perspective

Current studies have suggested that genetic factors contribute to the development of schizophrenia and dysbindin-1 has been identified as one of the susceptibility genes [49]. The following accumulating evidences propose the contribution of dysbindin-1 to the pathogenesis of schizophrenia: (i) dysbindin-1 is highly expressed in the dorsolateral prefrontal cortex and hippocampus [28], identified as major regions that may be altered in schizophrenic patients [105]; (ii) multiple SNPs of *DTNBP1* are suggested to influence different psychiatric phenotypes [65,66,67]; (iii) postmortem brain studies have indicated reduced expression of both dysbindin-1 mRNA and protein in the brains of schizophrenic patients [22,75]; (iv) dysbindin-1-deficient mice displayed schizophrenia-like behaviors, especially the negative symptoms of schizophrenia [86,87]; (v) dysbindin-1 affects the release of dopamine and glutamate and the trafficking of neurotransmitter receptors [41,46]; and (vi) dysbindin-1 is involved in neurite outgrowth [24,59,93]. Cumulatively, these findings suggest that multiple brain neuronal processes in different combinations may be affected by dysbindin-1 protein expression or activity, in order to trigger schizophrenia. The evidences gathered so far indicate multiple cellular activities of dysbindin in neurons. It is quite clear that dysbindin enters the nucleus to regulate transcription and shuttle from the nucleus to the cytoplasm to assemble into various multi-subunit protein complexes (e.g., BLOC-1) regulating the cytoskeleton, trafficking and signaling pathways. Moreover, multiple biologically active forms of dysbindin-1 would control neurite outgrowth and dendritic spine maturation during neuronal differentiation and, in mature neurons, the biogenesis and release of synaptic vesicles at pre-synaptic terminals as well as the down-regulation of neurotransmitter receptors at postsynaptic terminals. Thus, it is imperative to further expand our knowledge of multiple dysbindin-1 interacting protein candidates to schizophrenia disease susceptibility. A focus only on dysbindin-1 protein is unlikely to unravel this complex disease. Dysbindin-1 may confer its susceptibility to schizophrenia through its impact on dopaminergic and glutamatergic neurotransmission, which link to other neurochemical brain pathways. We also propose that dysbindin-1 might be a potential therapeutic target for the treatment of this schizophrenia. The mechanism(s) by which dysbindin-1 affects dopaminergic and glutamatergic signaling, and its effects on the different receptor subtypes and downstream molecules need further investigations. It is also important to examine the impact of environmental stress on the expression of dysbindin-1 levels in both animal models and patients. Finally, the effects of antipsychotics on the expression of dysbindin-1 are not fully understood and need to be further explored.

In conclusion, dysbindin-1 plays a broad role in the etiology of schizophrenia. Enhancing the level or activity of dysbindin-1 in the brain might be beneficial for the treatment of schizophrenia. However, this disorder occurs through a complicated interaction of multiple biochemical, neurochemical, genetic and environmental risk factors of a poorly understood pathology. Under these circumstances, it is therefore important to clarify dysbindin-1-mediated neuronal functions and to develop therapeutic molecules and modalities based on this target.

## References

[1] Wang H., Farhan M., Xu J., Lazarovici P., Zheng W. (2017). The involvement of darpp-32 in the pathophysiology of schizophrenia. Oncotarget.

[2] Zheng W., Wang H., Zeng Z., Lin J., Little P.J., Srivastava L.K., Quirion R. (2012). The possible role of the akt signaling pathway in schizophrenia. Brain Res..

[3] Kantrowitz J.T. (2017). Managing negative symptoms of schizophrenia: How far have we come?. CNS Drugs.

[4] Schooler N.R., Buchanan R.W., Laughren T., Leucht S., Nasrallah H.A., Potkin S.G., Abi-Saab D., Berardo C.G., Bugarski-Kirola D., Blaettler T. (2015). Defining therapeutic benefit for people with schizophrenia: Focus on negative symptoms. Schizophr. Res..

[5] Li P., Snyder G.L., Vanover K.E. (2016). Dopamine targeting drugs for the treatment of schizophrenia: Past, present and future. Curr. Top. Med. Chem..

[6] Matsumoto M., Walton N.M., Yamada H., Kondo Y., Marek G.J., Tajinda K. (2017). The impact of genetics on future drug discovery in schizophrenia. Expert. Opin. Drug Discov..

[7] Davis J., Eyre H., Jacka F.N., Dodd S., Dean O., McEwen S., Debnath M., McGrath J., Maes M., Amminger P. (2016). A review of vulnerability and risks for schizophrenia: Beyond the two hit hypothesis. Neurosci. Biobehav. Rev..

[8] Attademo L., Bernardini F., Garinella R., Compton M.T. (2017). Environmental pollution and risk of psychotic disorders: A review of the science to date. Schizophr. Res..

[9] Xu T., Wang Y., Li Z., Huang J., Lui S.S., Tan S.P., Yu X., Cheung E.F., He M.G., Ott J. (2016). Heritability and familiality of neurological soft signs: Evidence from healthy twins, patients with schizophrenia and non-psychotic first-degree relatives. Psychol. Med..

[10] Owens S.F., Picchioni M.M., Rijsdijk F.V., Stahl D., Vassos E., Rodger A.K., Collier D.A., Murray R.M., Toulopoulou T. (2011). Genetic overlap between episodic memory deficits and schizophrenia: Results from the maudsley twin study. Psychol. Med..

[11] Kendler K.S., Diehl S.R. (1993). The genetics of schizophrenia: A current, genetic-epidemiologic perspective. Schizophr. Bull..

[12] Riley B., Kendler K.S. (2006). Molecular genetic studies of schizophrenia. Eur. J. Hum. Genet..

[13] Cardno A.G., Gottesman I.I. (2000). Twin studies of schizophrenia: From bow-and-arrow concordances to star wars mx and functional genomics. Am. J. Med. Genet..

[14] Tang J., Fan Y., Li H., Xiang Q., Zhang D.F., Li Z., He Y., Liao Y., Wang Y., He F. (2017). Whole-genome sequencing of monozygotic twins discordant for schizophrenia indicates multiple genetic risk factors for schizophrenia. J. Genet. Genom..

[15] Owen M.J., Williams N.M., O’Donovan M.C. (2004). Dysbindin-1 and schizophrenia: From genetics to neuropathology. J. Clin. Investig..

[16] Prats C., Arias B., Moya-Higueras J., Pomarol-Clotet E., Parellada M., Gonzalez-Pinto A., Peralta V., Ibanez M.I., Martin M., Fananas L. (2017). Evidence of an epistatic effect between dysbindin-1 and neuritin-1 genes on the risk for schizophrenia spectrum disorders. Eur. Psychiatry.

[17] Donohoe G., Morris D.W., Clarke S., McGhee K.A., Schwaiger S., Nangle J.M., Garavan H., Robertson I.H., Gill M., Corvin A. (2007). Variance in neurocognitive performance is associated with dysbindin-1 in schizophrenia: A preliminary study. Neuropsychologia.

[18] Markov V., Krug A., Krach S., Jansen A., Eggermann T., Zerres K., Stocker T., Shah N.J., Nothen M.M., Treutlein J. (2010). Impact of schizophrenia-risk gene dysbindin 1 on brain activation in bilateral middle frontal gyrus during a working memory task in healthy individuals. Hum. Brain Mapp..

[19] Yuan Q., Yang F., Xiao Y., Tan S., Husain N., Ren M., Hu Z., Martinowich K., Ng J.S., Kim P.J. (2016). Regulation of brain-derived neurotrophic factor exocytosis and gamma-aminobutyric acidergic interneuron synapse by the schizophrenia susceptibility gene dysbindin-1. Biol. Psychiatry.

[20] Shintani N., Onaka Y., Hashimoto R., Takamura H., Nagata T., Umeda-Yano S., Mouri A., Mamiya T., Haba R., Matsuzaki S. (2014). Behavioral characterization of mice overexpressing human dysbindin-1. Mol. Brain.

[21] Petit E.I., Michalak Z., Cox R., O’Tuathaigh C.M., Clarke N., Tighe O., Talbot K., Blake D., Joel J., Shaw A. (2017). Dysregulation of specialized delay/interference-dependent working memory following loss of dysbindin-1a in schizophrenia-related phenotypes. Neuropsychopharmacoloy.

[22] Talbot K., Eidem W.L., Tinsley C.L., Benson M.A., Thompson E.W., Smith R.J., Hahn C.G., Siegel S.J., Trojanowski J.Q., Gur R.E. (2004). Dysbindin-1 is reduced in intrinsic, glutamatergic terminals of the hippocampal formation in schizophrenia. J. Clin. Investig..

[23] Weickert C.S., Straub R.E., McClintock B.W., Matsumoto M., Hashimoto R., Hyde T.M., Herman M.M., Weinberger D.R., Kleinman J.E. (2004). Human dysbindin (*DTNBP1*) gene expression in normal brain and in schizophrenic prefrontal cortex and midbrain. Arch. Gen. Psychiatry.

[24] Ghiani C.A., Starcevic M., Rodriguez-Fernandez I.A., Nazarian R., Cheli V.T., Chan L.N., Malvar J.S., de Vellis J., Sabatti C., Dell’Angelica E.C. (2010). The dysbindin-containing complex (BLOC-1) in brain: Developmental regulation, interaction with SNARE proteins and role in neurite outgrowth. Mol. Psychiatry.

[25] Ma X., Fei E., Fu C., Ren H., Wang G. (2011). Dysbindin-1, a schizophrenia-related protein, facilitates neurite outgrowth by promoting the transcriptional activity of p53. Mol. Psychiatry.

[26] Papaleo F., Burdick M.C., Callicott J.H., Weinberger D.R. (2014). Epistatic interaction between comt and *DTNBP1* modulates prefrontal function in mice and in humans. Mol. Psychiatry.

[27] Ji Y., Yang F., Papaleo F., Wang H.X., Gao W.J., Weinberger D.R., Lu B. (2009). Role of dysbindin in dopamine receptor trafficking and cortical gaba function. Proc. Natl. Acad. Sci. USA.

[28] Tang J., LeGros R.P., Louneva N., Yeh L., Cohen J.W., Hahn C.G., Blake D.J., Arnold S.E., Talbot K. (2009). Dysbindin-1 in dorsolateral prefrontal cortex of schizophrenia cases is reduced in an isoform-specific manner unrelated to dysbindin-1 mrna expression. Hum. Mol. Genet..

[29] Wang H., Yuan Y., Zhang Z., Yan H., Feng Y., Li W. (2014). Dysbindin-1c is required for the survival of hilar mossy cells and the maturation of adult newborn neurons in dentate gyrus. J. Biol. Chem..

[30] Straub R.E., Jiang Y., MacLean C.J., Ma Y., Webb B.T., Myakishev M.V., Harris-Kerr C., Wormley B., Sadek H., Kadambi B. (2002). Genetic variation in the 6p22.3 gene *DTNBP1*, the human ortholog of the mouse dysbindin gene, is associated with schizophrenia. Am. J. Hum. Genet..

[31] Menke A., Jockusch H. (1991). Decreased osmotic stability of dystrophin-less muscle cells from the mdx mouse. Nature.

[32] Benson M.A., Newey S.E., Martin-Rendon E., Hawkes R., Blake D.J. (2001). Dysbindin, a novel coiled-coil-containing protein that interacts with the dystrobrevins in muscle and brain. J. Biol. Chem..

[33] Ito H., Morishita R., Shinoda T., Iwamoto I., Sudo K., Okamoto K., Nagata K. (2010). Dysbindin-1, wave2 and abi-1 form a complex that regulates dendritic spine formation. Mol. Psychiatry.

[34] Nazarian R., Starcevic M., Spencer M.J., Dell’Angelica E.C. (2006). Reinvestigation of the dysbindin subunit of bloc-1 (biogenesis of lysosome-related organelles complex-1) as a dystrobrevin-binding protein. Biochem. J..

[35] Falcon-Perez J.M., Starcevic M., Gautam R., Dell’Angelica E.C. (2002). Bloc-1, a novel complex containing the pallidin and muted proteins involved in the biogenesis of melanosomes and platelet-dense granules. J. Biol. Chem..

[36] Talbot K., Louneva N., Cohen J.W., Kazi H., Blake D.J., Arnold S.E. (2011). Synaptic dysbindin-1 reductions in schizophrenia occur in an isoform-specific manner indicating their subsynaptic location. PLoS ONE.

[37] Larimore J., Ryder P.V., Kim K.Y., Ambrose L.A., Chapleau C., Calfa G., Gross C., Bassell G.J., Pozzo-Miller L., Smith Y. (2013). Mecp2 regulates the synaptic expression of a dysbindin-bloc-1 network component in mouse brain and human induced pluripotent stem cell-derived neurons. PLoS ONE.

[38] Starcevic M., Dell’Angelica E.C. (2004). Identification of snapin and three novel proteins (blos1, blos2, and blos3/reduced pigmentation) as subunits of biogenesis of lysosome-related organelles complex-1 (bloc-1). J. Biol Chem..

[39] Dickman D.K., Davis G.W. (2009). The schizophrenia susceptibility gene dysbindin controls synaptic homeostasis. Science.

[40] Newell-Litwa K., Salazar G., Smith Y., Faundez V. (2009). Roles of bloc-1 and adaptor protein-3 complexes in cargo sorting to synaptic vesicles. Mol. Biol. Cell.

[41] Iizuka Y., Sei Y., Weinberger D.R., Straub R.E. (2007). Evidence that the bloc-1 protein dysbindin modulates dopamine d2 receptor internalization and signaling but not d1 internalization. J. Neurosci..

[42] Oyama S., Yamakawa H., Sasagawa N., Hosoi Y., Futai E., Ishiura S. (2009). Dysbindin-1, a schizophrenia-related protein, functionally interacts with the DNA-dependent protein kinase complex in an isoform-dependent manner. PLoS ONE.

[43] Xu Y., Sun Y., Ye H., Zhu L., Liu J., Wu X., Wang L., He T., Shen Y., Wu J.Y. (2015). Increased dysbindin-1b isoform expression in schizophrenia and its propensity in aggresome formation. Cell Discov..

[44] Hattori S., Murotani T., Matsuzaki S., Ishizuka T., Kumamoto N., Takeda M., Tohyama M., Yamatodani A., Kunugi H., Hashimoto R. (2008). Behavioral abnormalities and dopamine reductions in sdy mutant mice with a deletion in *DTNBP1*, a susceptibility gene for schizophrenia. Biochem. Biophys. Res. Commun..

[45] Chen X.W., Feng Y.Q., Hao C.J., Guo X.L., He X., Zhou Z.Y., Guo N., Huang H.P., Xiong W., Zheng H. (2008). *DTNBP1*, a schizophrenia susceptibility gene, affects kinetics of transmitter release. J. Cell Biol..

[46] Papaleo F., Yang F., Garcia S., Chen J., Lu B., Crawley J.N., Weinberger D.R. (2012). Dysbindin-1 modulates prefrontal cortical activity and schizophrenia-like behaviors via dopamine/d2 pathways. Mol. Psychiatry.

[47] Metzinger L., Blake D.J., Squier M.V., Anderson L.V., Deconinck A.E., Nawrotzki R., Hilton-Jones D., Davies K.E. (1997). Dystrobrevin deficiency at the sarcolemma of patients with muscular dystrophy. Hum. Mol. Genet..

[48] Selemon L.D., Zecevic N. (2015). Schizophrenia: A tale of two critical periods for prefrontal cortical development. Transl. Psychiatry.

[49] Soma M., Wang M., Suo S., Ishiura S. (2014). Dysbindin-1, a schizophrenia-related protein, interacts with hdac3. Neurosci. Lett..

[50] Fu C., Chen D., Chen R., Hu Q., Wang G. (2015). The schizophrenia-related protein dysbindin-1a is degraded and facilitates nf-kappa b activity in the nucleus. PLoS ONE.

[51] Bennett A.O.M. (2008). Dual constraints on synapse formation and regression in schizophrenia: Neuregulin, neuroligin, dysbindin, disc1, musk and agrin. Aust. N. Z. J. Psychiatry.

[52] Fei E., Ma X., Zhu C., Xue T., Yan J., Xu Y., Zhou J., Wang G. (2010). Nucleocytoplasmic shuttling of dysbindin-1, a schizophrenia-related protein, regulates synapsin i expression. J. Biol. Chem..

[53] Cha D.S., Kudlow P.A., Baskaran A., Mansur R.B., McIntyre R.S. (2014). Implications of epigenetic modulation for novel treatment approaches in patients with schizophrenia. Neuropharmacology.

[54] Boersma M.C., Dresselhaus E.C., De Biase L.M., Mihalas A.B., Bergles D.E., Meffert M.K. (2011). A requirement for nuclear factor-kappab in developmental and plasticity-associated synaptogenesis. J. Neurosci..

[55] Roussos P., Katsel P., Davis K.L., Giakoumaki S.G., Lencz T., Malhotra A.K., Siever L.J., Bitsios P., Haroutunian V. (2013). Convergent findings for abnormalities of the nf-kappab signaling pathway in schizophrenia. Neuropsychopharmacology.

[56] Sochocka M., Diniz B.S., Leszek J. (2016). Inflammatory response in the CNS: Friend or foe?. Mol. Neurobiol..

[57] Mattson M.P., Culmsee C., Yu Z., Camandola S. (2000). Roles of nuclear factor kappab in neuronal survival and plasticity. J. Neurochem..

[58] Greenhill S.D., Juczewski K., de Haan A.M., Seaton G., Fox K., Hardingham N.R. (2015). Adult cortical plasticity depends on an early postnatal critical period. Science.

[59] Lee S.A., Kim S.M., Suh B.K., Sun H.Y., Park Y.U., Hong J.H., Park C., Nguyen M.D., Nagata K., Yoo J.Y. (2015). Disrupted-in-schizophrenia 1 (disc1) regulates dysbindin function by enhancing its stability. J. Biol. Chem..

[60] Talbot K., Cho D.S., Ong W.Y., Benson M.A., Han L.Y., Kazi H.A., Kamins J., Hahn C.G., Blake D.J., Arnold S.E. (2006). Dysbindin-1 is a synaptic and microtubular protein that binds brain snapin. Hum. Mol. Genet..

[61] Ilardi J.M., Mochida S., Sheng Z.H. (1999). Snapin: A snare-associated protein implicated in synaptic transmission. Nat. Neurosci..

[62] Feng Y.Q., Zhou Z.Y., He X., Wang H., Guo X.L., Hao C.J., Guo Y., Zhen X.C., Li W. (2008). Dysbindin deficiency in sandy mice causes reduction of snapin and displays behaviors related to schizophrenia. Schizophr. Res..

[63] Farrell M.S., Werge T., Sklar P., Owen M.J., Ophoff R.A., O’Donovan M.C., Corvin A., Cichon S., Sullivan P.F. (2015). Evaluating historical candidate genes for schizophrenia. Mol. Psychiatry.

[64] Schwab S.G., Knapp M., Mondabon S., Hallmayer J., Borrmann-Hassenbach M., Albus M., Lerer B., Rietschel M., Trixler M., Maier W. (2003). Support for association of schizophrenia with genetic variation in the 6p22.3 gene, dysbindin, in sib-pair families with linkage and in an additional sample of triad families. Am. J. Hum. Genet..

[65] Cheah S.Y., Lawford B.R., Young R.M., Morris C.P., Voisey J. (2015). Dysbindin (*DTNBP1*) variants are associated with hallucinations in schizophrenia. Eur. Psychiatry J. Assoc. Eur. Psychiatr..

[66] Bakanidze G., Brandl E.J., Hutzler C., Aurass F., Onken S., Rapp M.A., Puls I. (2016). Association of dystrobrevin-binding protein 1 polymorphisms with sustained attention and set-shifting in schizophrenia patients. Neuropsychobiology.

[67] Baek J.H., Kim J.S., Ryu S., Oh S., Noh J., Lee W.K., Park T., Lee Y.S., Lee D., Kwon J.S. (2012). Association of genetic variations in *DTNBP1* with cognitive function in schizophrenia patients and healthy subjects. Am. J. Med. Genet. B Neuropsychiatr. Genet..

[68] Trost S., Platz B., Usher J., Scherk H., Wobrock T., Ekawardhani S., Meyer J., Reith W., Falkai P., Gruber O. (2013). The *DTNBP1* (dysbindin-1) gene variant rs2619522 is associated with variation of hippocampal and prefrontal grey matter volumes in humans. Eur. Arch. Psychiatry Clin. Neurosci..

[69] Voisey J., Swagell C.D., Hughes I.P., Connor J.P., Lawford B.R., Young R.M., Morris C.P. (2010). A polymorphism in the dysbindin gene (*DTNBP1*) associated with multiple psychiatric disorders including schizophrenia. Behav. Brain Funct..

[70] Schizophrenia Working Group of the Psychiatric Genomics Consortium (2014). Biological insights from 108 schizophrenia-associated genetic loci. Nature.

[71] Cox M.M., Tucker A.M., Tang J., Talbot K., Richer D.C., Yeh L., Arnold S.E. (2009). Neurobehavioral abnormalities in the dysbindin-1 mutant, sandy, on a c57bl/6j genetic background. Genes Brain Behav..

[72] Saggu S., Cannon T.D., Jentsch J.D., Lavin A. (2013). Potential molecular mechanisms for decreased synaptic glutamate release in dysbindin-1 mutant mice. Schizophr. Res..

[73] Bhardwaj S.K., Ryan R.T., Wong T.P., Srivastava L.K. (2015). Loss of dysbindin-1, a risk gene for schizophrenia, leads to impaired group 1 metabotropic glutamate receptor function in mice. Front. Behav. Neurosci..

[74] Papaleo F., Weinberger D.R. (2011). Dysbindin and schizophrenia: It’s dopamine and glutamate all over again. Biol. Psychiatry.

[75] Kendler K.S. (2004). Schizophrenia genetics and dysbindin: A corner turned?. Am. J. Psychiatry.

[76] Talbot K. (2009). The sandy (sdy) mouse: A dysbindin-1 mutant relevant to schizophrenia research. Prog. Brain Res..

[77] Takao K., Toyama K., Nakanishi K., Hattori S., Takamura H., Takeda M., Miyakawa T., Hashimoto R. (2008). Impaired long-term memory retention and working memory in sdy mutant mice with a deletion in *DTNBP1*, a susceptibility gene for schizophrenia. Mol. Brain.

[78] Coyle J.T. (2017). Schizophrenia: Basic and clinical. Adv. Neurobiol..

[79] Carr G.V., Jenkins K.A., Weinberger D.R., Papaleo F. (2013). Loss of dysbindin-1 in mice impairs reward-based operant learning by increasing impulsive and compulsive behavior. Behav. Brain Res..

[80] Braff D.L., Geyer M.A., Swerdlow N.R. (2001). Human studies of prepulse inhibition of startle: Normal subjects, patient groups, and pharmacological studies. Psychopharmacology.

[81] Karlsgodt K.H., Robleto K., Trantham-Davidson H., Jairl C., Cannon T.D., Lavin A., Jentsch J.D. (2011). Reduced dysbindin expression mediates n-methyl-d-aspartate receptor hypofunction and impaired working memory performance. Biol. Psychiatry.

[82] Abi-Dargham A. (2017). A dual hit model for dopamine in schizophrenia. Biol. Psychiatry.

[83] Weinstein J.J., Chohan M.O., Slifstein M., Kegeles L.S., Moore H., Abi-Dargham A. (2017). Pathway-specific dopamine abnormalities in schizophrenia. Biol. Psychiatry.

[84] Horga G., Cassidy C.M., Xu X., Moore H., Slifstein M., Van Snellenberg J.X., Abi-Dargham A. (2016). Dopamine-related disruption of functional topography of striatal connections in unmedicated patients with schizophrenia. JAMA Psychiatry.

[85] Schmieg N., Rocchi C., Romeo S., Maggio R., Millan M.J., Mannoury la Cour C. (2016). Dysbindin-1 modifies signaling and cellular localization of recombinant, human d(3) and d(2) receptors. J. Neurochem..

[86] Tang T.T., Yang F., Chen B.S., Lu Y., Ji Y., Roche K.W., Lu B. (2009). Dysbindin regulates hippocampal ltp by controlling nmda receptor surface expression. Proc. Natl. Acad. Sci. USA.

[87] Glen W.B., Horowitz B., Carlson G.C., Cannon T.D., Talbot K., Jentsch J.D., Lavin A. (2014). Dysbindin-1 loss compromises nmdar-dependent synaptic plasticity and contextual fear conditioning. Hippocampus.

[88] Jentsch J.D., Trantham-Davidson H., Jairl C., Tinsley M., Cannon T.D., Lavin A. (2009). Dysbindin modulates prefrontal cortical glutamatergic circuits and working memory function in mice. Neuropsychopharmacology.

[89] Gupta S., Kulhara P. (2010). What is schizophrenia: A neurodevelopmental or neurodegenerative disorder or a combination of both? A critical analysis. Indian J. Psychiatry.

[90] Lefebvre J.L., Sanes J.R., Kay J.N. (2015). Development of dendritic form and function. Annu. Rev. Cell Dev. Biol..

[91] Kubota K., Kumamoto N., Matsuzaki S., Hashimoto R., Hattori T., Okuda H., Takamura H., Takeda M., Katayama T., Tohyama M. (2009). Dysbindin engages in c-jun n-terminal kinase activity and cytoskeletal organization. Biochem. Biophys. Res. Commun..

[92] Kamiya A., Tomoda T., Chang J., Takaki M., Zhan C., Morita M., Cascio M.B., Elashvili S., Koizumi H., Takanezawa Y. (2006). Disc1-ndel1/nudel protein interaction, an essential component for neurite outgrowth, is modulated by genetic variations of disc1. Hum. Mol. Genet..

[93] Zhang J., Yan W., Chen X. (2006). P53 is required for nerve growth factor-mediated differentiation of pc12 cells via regulation of trka levels. Cell Death Differ..

[94] Ryder P.V., Faundez V. (2009). Schizophrenia: The “bloc” may be in the endosomes. Sci. Signal.

[95] Mead C.L., Kuzyk M.A., Moradian A., Wilson G.M., Holt R.A., Morin G.B. (2010). Cytosolic protein interactions of the schizophrenia susceptibility gene dysbindin. J. Neurochem..

[96] Sudhof T.C. (2004). The synaptic vesicle cycle. Annu. Rev. Neurosci..

[97] Hikita T., Taya S., Fujino Y., Taneichi-Kuroda S., Ohta K., Tsuboi D., Shinoda T., Kuroda K., Funahashi Y., Uraguchi-Asaki J. (2009). Proteomic analysis reveals novel binding partners of dysbindin, a schizophrenia-related protein. J. Neurochem..

[98] Gokhale A., Hartwig C., Freeman A.H., Das R., Zlatic S.A., Vistein R., Burch A., Carrot G., Lewis A.F., Nelms S. (2016). The proteome of bloc-1 genetic defects identifies the arp2/3 actin polymerization complex to function downstream of the schizophrenia susceptibility factor dysbindin at the synapse. J. Neurosci..

[99] Kim I.H., Racz B., Wang H., Burianek L., Weinberg R., Yasuda R., Wetsel W.C., Soderling S.H. (2013). Disruption of arp2/3 results in asymmetric structural plasticity of dendritic spines and progressive synaptic and behavioral abnormalities. J. Neurosci..

[100] Rocca D.L., Amici M., Antoniou A., Blanco Suarez E., Halemani N., Murk K., McGarvey J., Jaafari N., Mellor J.R., Collingridge G.L. (2013). The small gtpase arf1 modulates arp2/3-mediated actin polymerization via pick1 to regulate synaptic plasticity. Neuron.

[101] Jia J.M., Hu Z., Nordman J., Li Z. (2014). The schizophrenia susceptibility gene dysbindin regulates dendritic spine dynamics. J. Neurosci..

[102] Gokhale A., Mullin A.P., Zlatic S.A., Easley C.A., Merritt M.E., Raj N., Larimore J., Gordon D.E., Peden A.A., Sanyal S. (2015). The n-ethylmaleimide-sensitive factor and dysbindin interact to modulate synaptic plasticity. J. Neurosci..

[103] Gokhale A., Larimore J., Werner E., So L., Moreno-De-Luca A., Lese-Martin C., Lupashin V.V., Smith Y., Faundez V. (2012). Quantitative proteomic and genetic analyses of the schizophrenia susceptibility factor dysbindin identify novel roles of the biogenesis of lysosome-related organelles complex 1. J. Neurosci..

[104] Han M.H., Hu Z., Chen C.Y., Chen Y., Gucek M., Li Z., Markey S.P. (2014). Dysbindin-associated proteome in the p2 synaptosome fraction of mouse brain. J. Proteome Res..

[105] Guillozet-Bongaarts A.L., Hyde T.M., Dalley R.A., Hawrylycz M.J., Henry A., Hof P.R., Hohmann J., Jones A.R., Kuan C.L., Royall J. (2014). Altered gene expression in the dorsolateral prefrontal cortex of individuals with schizophrenia. Mol. Psychiatry.

